# Supplementary material for: The adaptive immune response to Trichuris in wild versus laboratory mice: An established model system in context
Source: PLoS Pathog. 2024 Apr 16;20(4):e1012119. doi: 10.1371/journal.ppat.1012119 (PMC11051619; doi:10.1371/journal.ppat.1012119)
Supplement: Attachment — Submitted filename: Letter in response to reviewers comments_FINAL.docx [file ppat.1012119.s015.docx]

*Dear Editor,*

*We thank you and the reviewers for their insightful and thorough comments on the manuscript entitled ‘The adaptive immune response to Trichuris in wild versus laboratory mice: An established model system in context’. We are delighted to hear that the content of the manuscript was perceived as relevant and timely. We thank the editor for reviewing the reviewer’s comments and highlighting the areas they felt we should attend to, including advising that no additional experimentation is required. Below, we have addressed each point in response to the reviewer’s comments.*

***Response to Reviewer 1***

*We thank reviewer 1 for their helpful review and the interesting and engaged comments they have made. Our response to each comment is below, with any new content indicated as appropriate. We have also taken into account the advice of the editor that no additional experimentation is required.*

While the research questions this manuscript attempts to address are interesting and relevant, it is difficult to assess the effects seen in wild mice in comparison to those in laboratory mice, as they are convoluted by multiple factors that were not excluded, including food sources and availability, stress from living in the wild (weather, predators…), continuous pregnancies of female mice. The authors acknowledge this and are making the point that this is one aspect they were trying to assess. Yet, I am somewhat concerned about how valid the conclusions from such studies can be. The genetics of wild mice are mixed. Further, the authors mention that wild mice were in many cases infected with additional helminths. The study also assesses age as a factor, by comparing juvenile to adult wild mice. But what were the parameters to determine the age of the wild mice? Are they reliable or arbitrary? (Eg. size or weight could be reduced due to food scarcity or additional environmental factors.) While the study has great potential, the conclusions would be much clearer if this multitude of factors could be controlled.

*The reviewer raises some important points. We outline our answers to each point below.*

*1. The validity of the wild to lab comparison: We used fully wild mice as a comparator precisely in order to have uncontrolled variation in multiple factors – both genetic and environmental. Laboratory model systems are per se aimed to model ‘wild’ humans or other animals living in their natural context. Whilst this means it is impossible to trace all variation back to their original source as the reviewer rightly points out, what it enables us is to get an understanding of how well an immunological model system matches and mimics a fully uncontrolled system despite all these factors. We agree this needs further comment and we have added a section in to our Discussion (lines 354-366) where we outline to motivation for the comparison and its limitations as below.*

Motivated by a desire to understand where the wild mouse cellular and cytokine responses fell in the context of typical laboratory based analyses of the cellular immune responses to whipworm infection, we compared cytokine profiles seen in *T. muris* infected C57BL/6 mice to those of the wild mice. We chose the C57BL/6 mouse for a number of reasons: C57BL/6 mice are the most widely used inbred strain of mouse in laboratory studies in general; it was the strain used to create the *Mus musculus* reference genome; there is a wealth of data concerning how it responds to *T. muris* infection; it permits the exploration of immune response to both a high dose and a low dose infection levels of whipworm. In the current study, in addition to genetics, we held constant many of the extrinsic and intrinsic factors such as sex, age, diet, which varied in the wild mouse population and each of which has been shown singularly to affect the immune response in laboratory studies (27-29). We acknowledge that this is a reductionist approach and creates an asymmetrical comparison, however the approach does allow a comparison to be made between the most commonly used laboratory mouse and a fully wild *Mus musculus* population.

*2. The parameters used to assess age. We considered very carefully how best to assess age in the wild mice and decided based on the data available that a multi-parameter proxy is most robust, acknowledging – as the reviewer pointed out - that single paramaters such as size and age can be variable due to other ecological factors. We believe our “maturity index” provides a sensible proxy for age in the absence of known birth date and have extended our text in the Methods (Lines 642 -658) to explain our rationales more fully. We are aware of DNA methylation assays as a new methodology for more precisely estimating age in wild animal populations (eg De Paoli-Iseppi R, Deagle BE, McMahon CR, Hindell MA, Dickinson JL, Jarman SN. Measuring Animal Age with DNA Methylation: From Humans to Wild Animals. Front Genet. 2017 Aug 17;8:106. doi: 10.3389/fgene.2017.00106. PMID: 28878806; PMCID: PMC5572392.). When this method becomes more affordable, this will be an important addition to the toolbox of wild animal research as we agree that age is an important host factor to consider.*

While age is typically estimated from dry eye lens mass in wild rodent populations, building on the formulae set out by Rowe *et al*. 1985 (71), we found this method to not be fully accurate on its own, as ages were calculated which were known to be impossible i.e. mice were known to be older than the calculated ages based on recapture data. In order to develop a useful and continuous proxy measure of age, a principal component analysis (PCA) was performed on cull samples, incorporating body mass, body length, tail length and dry paired eye lens mass, in order to provide a ‘maturity index’. PC1 explained 69.6% of variation in these samples and described uniform increases across all included metrics, and so PC1 scores were taken as maturity index values. In order to have an equivalent index for non-cull samples, another PCA was carried out across all samples, excluding eye lens mass. PC1 explained 69.3% of variation in this analysis. These maturity PCA scores were strongly correlated with the analogous scores from cull (Pearson correlation estimate 0.976, p<2.2e^-16^), meaning the longitudinal scores are a suitable proxy for the cull values which include eye lens mass data. Increases in longitudinal maturity index showed strong positive correlation with the time since an animal was first captured (Spearman correlation rho=0.394, p= 7.194e^-15^). The longitudinal maturity index was therefore taken as an appropriate measure of biological age when required for longitudinal analysis. Further PCA details and loadings are provided in Supporting Materials.

The authors may consider co-housing studies and/or controlled exposure studies, similar to many recent publications. For example, laboratory mice could be co-housed with eg. wild or pet store mice to introduce exposure to infections, whilst all other factors would still be controlled.

*The strength of the wild Mus musculus system lies in the fact that the mice live in a fully uncontrolled multi-variate environment, and so capture any natural variation at play in free-living populations. We believe that the co-housing and controlled exposure (semi-wild) studies mentioned above are powerful and complementary to our fully wild systems, and in the future would indeed allow us to tease apart contributing factors to any phenotype observed in the wild.*

To exclude merely gene-/strain specific effects, multiple laboratory mouse strains could be tested against wild or co-housed mice, rather than only C57BL/6. (As long as the strains do not succumb to T.m. infection.)
It is difficult to assess whether the different response to T.m. in wild mice as compared to lab C57BL/6 is due to previous and/or constant exposure to this and/or other helminths, and/or due to microbial diversification/normalization/infection history. Experiments in Figure 2 are along those lines but could be extended. To shed further light on this, laboratory mice could be exposed to T.m. multiple times or chronically, and at distinct doses. This could be compared to T.m. infections in wild mouse co-housed laboratory mice. The wild mice could be treated for helminths prior to co-housing to exclude previous helminth exposure as a factor.

*Please see response above regarding the wild to lab comparison (Point 1) and the amends we have made to our discussion (lines 354-366) to capture this conversation.*

*In addition the reviewer makes some other useful suggestions. Based on the results from our study, it would indeed be interesting to use a variety of different inbred strains of laboratory mouse, or an outbred strain of laboratory mouse or the Collaborative Cross (Threadgill DW, Miller DR, Churchill GA, de Villena FP. The collaborative cross: a recombinant inbred mouse population for the systems genetic era. ILAR J. 2011;52(1):24-31. doi: 10.1093/ilar.52.1.24. PMID: 214118550). We could use different infection regimes including a “trickle” style infection that may better reflect levels of exposure in the wild. These are all excellent suggestions that we can build on now that we have quantified the cellular immune responses in the reference strain of the C57BL/6 mouse which we still feel is a useful starting point since it is the most widely used strain. We have expanded on future directions in the discussion to reflect these possibilities (lines 430-436).*

Questions arising from the current study include how much of the variation in immune response is genetically driven. Teasing apart genetic versus environmental drivers could be approached by genetic analysis of the wild mice and a comparison of immune responses across a variety of inbred and outbred laboratory strains in relation to the wild mice. Further, given that exposure to infection is likely repetitive in the wild, trickle infections in laboratory animals (25) would warrant a comparison. Finally, ecoimmunological studies such as the one we present here, serve as hypothesis generators for interventional experiments in the wild, targeting e.g. the diet or the immune system itself, in order to provide mechanistic insights.

The study introduces a “gut responsiveness score”. While this is an interesting idea, it does not seem to be an established method and may need to be validated. It is unclear whether the scoring of mice was blinded or not. Since this score is an unconventional analysis method, a statistician may need to be consulted to determine whether the conclusions drawn are valid.

*The scoring of mice was carried out in a blinded fashion.*

*The scoring system is based upon standard methodologies used in the context of inflammatory bowel disease, for which we have now inserted references (line 607; Erben et al . Int J Clin Exp Pathol. 2014;7(8):4557-76; Geboes et al Gut. 2000;47(3):404-90).*

*We ourselves have deployed scoring systems previously in the context of T. muris infection (now referenced in line 609; Bhardwaj et al World J Gastroenterol. 2014 Feb 21;20(7):1797-806). We have further extended our methods section to include more detail on our use of the 4 gut responsiveness measures. Thus we now state (Lines 632-636):*

A PCA was used in place of an additive multiparameter score in order to give a scaled, and less dimensional measure of gut responsiveness i.e. to remove the influence of any negative relationships between parameters, and leave only a measure of increasing responsiveness across parameters. These PC1 scores are highly correlated with the additive multiparameter score (Spearman’s rho=0.87, p<2.2e^-16^).

The cytokine responses in wild mice seemed reduced compared to laboratory mice, even though the wild mice had a larger CD4 Tem compartment. This could be due to clonal expansion of T cells in wild mice which had been exposed to a multitude of infections. Further, previous work has shown enhanced polarization towards Th1 responses in a “dirty mouse” setting. The effects seen in the current study may be a reflection of that. However, to add a more novel aspect, it could be interesting to assess whether there is a difference in wild vs laboratory mouse Th2 cells at the cellular level, for example by looking at the MFI of cytokine producing CD4 T cells in wild vs laboratory for each cytokine by flow cytometry. Is there an actual change in response by Th2 cells per se?

*We agree with the reviewer’s suggestion that the enriched T_EM_ in the wild mice is likely due to more exposure, with only a fraction of the T_EM_ likely being T. muris specific. Our main reason for analysing the CD4+ T_em_ compartment of wild mice was to check whether the lower levels of cytokines were associated with fewer T_EM_. As higher T_EM_ proportions in wild mice have been previously reported, we decided against focussing on this in the discussion, but have now added a reference in this respect in the results text (line 236):*

In fact, wild mouse mLNs harboured significantly enlarged populations of T_EM_ compared to laboratory mice, as has been previously reported in other wild mouse populations and is considered likely a result of repeated exposure to immunogenic antigens.

*The study the reviewer mentions, which we believe to be Leung et al., 2018, revealed an enhanced polarisation to Th1 in genetically defined lab mice released into a semi-wild system, and complements our work by focussing on controlled genetics but a rapid change in environment from a controlled to an uncontrolled setting. However, in our fully wild setting we show lower cytokine levels per se and a mixed Th1/Th2 response. We agree that it would be very valuable to look at cytokine levels on a cellular level. We would have liked to have this information, however this was technically not feasible, given that intracellular cytokine staining needs to be acquired within a day of stimulation and staining, and we did not have flow cytometry facilities available on the island.*

***Response to Reviewer 2***

*We thank reviewer 2 for their helpful review and the interesting and engaged comments they have made. Our response to each comment is below, with any new content indicated as appropriate. We have also taken into account the advice of the editor that no additional experimentation is required.*

The study is strong overall, but requires better explanation of methods and inferences to clarify the manuscript. Several suggestions for revision to improve the manuscript are as follows:

1) The authors need to explain more clearly how the 2 pieces of the study fit together. More explanation is needed because the analyses focus on asymmetrical comparisons, in terms of sample sizes in lab vs wild as well as various factors potentially affecting immune function: only male mice of a particular genetic background and age range, and given particular doses of worms, were studied in the lab, whereas both sexes, various genetic backgrounds, and all ages/exposure histories were studied in the wild. This raises many questions. For example, in Fig 2G, were young adult males in lab and wild actually equivalent, such that the lab experiment simply captured a fraction of the range of variation among wild individuals?

Relatedly, why was Aug-Dec selected for the wild study season, and were the mice breeding at the time? The answers to these questions would shed further light on the causes of the lab-wild divergence.

*We agree that the comparison between wild mice and laboratory C57BL/6 mice is asymmetrical, yet we believe it is a very important comparison to make given that controlled laboratory studies are used to model uncontrolled populations, whether humans or animals. Please see our response to reviewer 1 and addition to our Discussion lines 354-366 and 430-436) to address this asymmetry more clearly in the manuscript.*

*Regarding the specific question of whether young males in the wild matched the young males used in the laboratory; our analysis does not give evidence that this is the case. The redundancy analysis returned sex as a non-significant factor in explaining Trichuris-specific cytokine responses. Increasing maturity index significantly explained enhanced and more Th2-biased cytokine production; young male wild mice would therefore not match with the strong Th1/Th2 mixed response seen in most laboratory groups analysed.*

*With respect to selecting trapping season, August to December was selected for this study as the population undergoes a yearly boom-and-crash cycle (see Triggs, Journal of Zoology 1991, doi.org/10.1111/j.1469-7998.1991.tb03828.x), with sufficient numbers of animals captured and recaptured only present between these months on the island. We have added the following line in the Materials and Methods section, under ‘Mice’, lines 452-454:*

August to December was chosen as trapping season as the mice undergo a yearly population crash over winter with sufficient trapping power only returning around August each year.

*In addition, we include below a representative graph of capture, mark and recapture rates across months on the Fluke Street grid for a visual representation of this boom-and-crash cycle.*

**Month**

**Capture (n mice)**

*With respect to whether the mice were breeding during our trapping seasons: We trapped juveniles throughout the year (apart from March). We do not have information on juvenile to adult ratios in January/February due to the island being closed down during this time, however, the ratio of juvenile to adult peaks in August and declines towards the winter.*

2) The authors should provide more explanatory context for their most interesting finding, that only in older mice was high worm burden associated with Th1 and low burden with Th2 immune responses. The criteria for dividing younger versus older were not clear (around Line 244), but suggested that the authors perhaps used the median estimated age to define the groups?? In any case, the authors should justify their statistical modelling approach. For example, why model the cohorts separately (as in S9 Table) rather than using an Age class*worm burden interaction term, to understand interacting effects of age and worm burden on immune response? Furthermore, what is the age distribution among and within these age groups? As is, it is unclear if dividing individuals into two groups fully explains this relationship of immunoparasitology with age, or if this cohort distinction is meaningful.

*We have now clarified in the results text that the young and older age cohorts was* based on *median maturity index* *(see Suppl. Fig S9 for cutoff choice) (Line 264). There is a precedent in the human whipworm literature for the use of age cohorts when analysing cytokine responses (eg Faulkner et al J Infect Dis. 2002 Mar 1;185(5):665-72. ; Turner et al J Infect Dis. 2008 Apr 15;197(8):1204-12). We might expect an age related change to be more step-wise so rather than using a continuous variable – not necessarily a linear relationship i.e. not caught with age class*worm burden interaction. Models using a range of maturity cut-off values were performed, and using a Maturity Index value of 0 showed the strongest old-cohort-specific association between Trichuris and cytokine levels, and the most equitable likelihood values across models from both cohorts.*

*We have now included a supplementary figure detailing the justification for using the median maturity index as cut-off for robust statistical analyses and added to our Methods (lines 686-692)*

Age cohorts were chosen as we expected age-related changes in Trichuris immunity to be present as a sharp, qualitative shift, rather than a gradual linear relationship, with the cohort cutoffs selected to provide roughly equal group sizes for optimal model fitting, in lieu of a reliable measure of true age with which to establish a biologically-informed cutoff value (age-cutoff comparison and justification shown in Suppl. Fig S9). There were 124 mice designated to the ‘young’ cohort and 142 to the ‘old’ cohort. Group sizes were not identical as the median Maturity Index value of 0 was established from the whole cross-sectional dataset.

*We of course do not know the actual ages of the wild mice but now show the distribution within the age groups as pasted below.*

3) In general, the level of explanation of methods is lacking. For example, the results section should include a more descriptive preamble of the methods, including statistical methods, to help readers understand what the results mean. In particular, novel metrics proposed, like—“maturity index” and “gut responsiveness,”—need main text explanation from the first appearance, so that readers can immediately follow what the terms mean. The explanations not only should appear earlier, but they need more detail. For example, when maturity index is finally explained in Line 574: Why is PC1 taken as a maturity index (what are the loadings, etc)? And what is the rationale for choosing this metric? Do larger PC1 values correlate with mice known to be older? Please include these data in the supplementary data if so. Furthermore, the authors should briefly explain the statistical analyses used in each section, prior to presenting the results, because as they stand the reported stats are hard to judge.

*We apologise for our lack of detail in our Methods. We have now added detail in to our methods in terms of the statistical methods and explained the maturity index and gut responsiveness.*

*Methods:*

(Lines 646- 659) In order to develop a useful and continuous proxy measure of age, a principal component analysis (PCA) was performed on cull samples, incorporating body mass, body length, tail length and dry paired eye lens mass, in order to provide a ‘maturity index’. PC1 explained 69.6% of variation in these samples and described uniform increases across all included metrics, and so PC1 scores were taken as maturity index values. In order to have an equivalent index for non-cull samples, another PCA was carried out across all samples, excluding eye lens mass. PC1 explained 69.3% of variation in this analysis. These maturity PCA scores were strongly correlated with the analogous scores from cull (Pearson correlation estimate 0.976 , p<2.2e-16), meaning the longitudinal scores are a suitable proxy for the cull values which include eye lens mass data. Increases in longitudinal maturity index showed strong positive correlation with the time since an animal was first captured (Spearman correlation p= 7.194e-15). The longitudinal maturity index was therefore taken as an appropriate measure of biological age when required for longitudinal analysis. Further PCA details and loadings are provided in Supporting Materials.

*Results:*

(Line 256-158) Using a logistic regression model and a proxy for age, derived from a primary principal component capturing multiple aspects of morphometry expected to increase with biological age (hereafter ‘maturity index’, see methods for further details).

(Line 177-181) To assess the health consequences of harbouring *T. muris* at the organ level, we generated a proxy for gut architecture disturbance comprised of the primary principal component of four histological measures (level of immune cell infiltration, location of infiltration, goblet cells per crypt, and crypt length) which we termed ‘gut responsiveness’ (see methods and S5 Fig for further details).

*Methods:*

*Gut responsiveness: We have added references to scoring systems for Inflammatory Bowel Disease and gut inflammatory models which formed the basis of the ‘Gut responsiveness score’ (line 607), as well as explaining the use of PC1 in more detail:*

(Lines 632-636) A PCA was used in place of an additive multiparameter score in order to give a scaled, and less dimensional measure of gut responsiveness i.e. to remove the influence of any negative relationships between parameters, and leave only a measure of increasing responsiveness across parameters. These PC1 scores are highly correlated with the additive multiparameter score (Spearman’s rho=0.87, p<2.2e^-16^).

*Maturity index:*

(Line 649) PC1 explained 69.6% of variation in these samples and described uniform increases across all included metrics.

(Lines 652-657) These maturity PCA scores were strongly correlated with the analogous scores from cull (Pearson correlation estimate 0.976, p<2.2e^-16^), meaning the longitudinal scores are a suitable proxy for the cull values which include eye lens mass data. Increases in longitudinal maturity index showed strong positive correlation with the time since an animal was first captured (Spearman correlation rho=0.394, p= 7.194e^-15^).

*We have also included more detail on the statistics used in each results section to facilitate the reader’s interpretation of the results:*

(Line 202) Using a Kruskal-Wallis test followed by a Dunn’s test with Bonferroni adjustment to compare PC1 and PC2 across experimental groups*,…(relating to Figure 2)*

(Line 218) Hypothesising that the proportion of CD4+ T cells, Foxp3^+^ regulatory T cells, T_EM_, T-bet^+^ T_EM_ and GATA3^+^ T_EM_ may affect the observed variation in cytokine expression, we used these markers as predictors in a generalised linear models to explain either PC1 or PC2. *(relating to Figure 3)*

(Line 231) …, using a Kruskal-Wallis test followed by a Dunn’s test with Bonferroni adjustment *(relating to Figure 3c)*

Additional minor suggestions for clarification, in order of appearance by line number (L):
L156: Perhaps extract the odds ratio from the linear regression estimate for a more intuitive comparison of likelihood to be infected.

*Thank you for the suggestion. This has now been amended accordingly (now line 159). Also, we have noticed that we accidentally wrote ‘linear regression’ in this sentence while the regression was a logistic regression as stated correctly in the previous sentence. We have corrected this too.*

(logistic regression odds ratio=1.446 , *p*=2.13x10^-4^)

L160: The data presented indicates low worm burdens, but not necessarily chronicity. While low worm burdens are suggestive of chronic infections, it should be presented as a possibility rather than a fact based on the study’s data. A brief explanation with a citation that justifies this interpretation could be sufficient. See also L301 about chronic infection.

*In the current manuscript we are using the word “chronic” with the following definition which we have added to our Methods (lines 585- 590), and defined briefly upon first use of the word “chronic” in the Abstract (Line 29).*

Chronic infections of soil-transmitted helminths, which we define as establishment of adult, fecund worms, impose significant health burdens on humans, livestock and wildlife, with limited treatment success.

Historically in laboratory based studies, the word “chronic” has been defined as an infection maintained in the host until the egg laying, mature adult worms are present (69, 70) i.e. the life cycle would be maintained in a natural population. Thus, the animal is susceptible to infection. Any host that expels the parasite prior to the development of the adult stage (around 32 days post infection) is deemed resistant and unable to harbour a chronic infection*.*

L165: The authors describe a fascinatingly high prevalence of Calodium hepaticum and thus polyparasitism in the wild. Does this suggest that the wild mice routinely engage in cannibalism (at least scavenging meat from dead conspecifics), or is some other mode of transmission of that parasite predominant on the island? If cannibalism is likely, what are the wider implications for nutritional plane in wild mice? And is C. hepaticum expected to immunomodulate the host?

*The presence of Calodium at such a high prevalence is interesting. We have added our thoughts in terms of the main infection route of this tissue dwelling nematode on the Isle of May to the results section (lines 171-176). As with all parasitic nematodes, it is likely that Calodium may modulate host immunity. With limited diagnostic assays available for Calodium we could unfortunately not provide more detail on this infection in this study. We have however added a comment about potential immunomodulatory effects as detailed below.*

*C. hepaticum* can be transmitted via necrophagy, or through ingestion of embryonated eggs in the environment following decay of an infected host (33). There is no explicit evidence, nor anecdotal report of necrophagy occurring in this population (previous studies found variation in sources of dietary nitrogen, but the source of this variation is unknown (34), and as such this parasite has been assumed to be transmitted through environmental ingestion in this instance. Whilst immunomodulation by *Calodium* is likely, we did not carry out further parasite-specific assays due to the scarcity of diagnostic tools for this parasite (35).

L170: The data seem normally distributed given the scatter, so why was this analyzed with a Kruskal-Wallis test?

*(New Line 183) Thank you for the comment. The Gut responsiveness scores are actually not normally distributed and hence we used the Kruskal-Wallis test.*

L191: Is this figure referring to 2G?

*We apologise for the mismatch in the figure and text, we have corrected the in-text reference from 2E through to 2G.*

L250: Are these longitudinal data from wild mice only? So, mice were not culled upon first capture? Please clarify the duration of trapping and any recapture rates.

*We apologise for any confusion. The longitudinal data is from wild mice only and these mice were not culled at first capture. At first capture mice were tagged, tail bled, faecal samples collected and morphometrics captured. Mice were then released. Mice were subsequently culled if recaptured during one of our culling sessions Nov-Dec 2018 and Sept-Dec 2019. We have extended our Methods section clarify:*

(Lines 453-467) August to December was chosen as trapping season as the mice undergo a yearly population crash over winter with sufficient trapping power only returning around August each year. Trapping at one grid (ML) was halted following the August 2019 trapping session, due to limitations in field worker numbers. There were a total of 14 trapping days at the ML grid, 30 at the LL grid and 31 at the FS grid (trapping effort detail provided in Suppl. Fig S13). From August to December each year, caught mice were checked for Passive Integrated Transponders (PITs), and were PIT tagged if none was detected. Basic morphometric measures (body mass, snout-vent body length and tail length) were taken using callipers and scales. A total of 492 mice were PIT tagged between August-November in 2018 and 2019. Mice captured from November to December in 2018 and from September to December 2019, and which had been previously trapped, were culled (n=214). Another 58 untagged mice caught during culling sessions were also culled, where fieldwork capacity allowed. All culled mice formed our ‘Cross-Sectional’ set (n=272), and were used for detailed immunological and histological analyses. Of these culled mice, 91 animals had been captured 30 days previously (recaptures) and so also entered into our ‘Longitudinal’ study, allowing us to investigate changes in individuals over time. Across the two years, overall recapture rate was 40% within one month.

L280: Which one is the cross-sectional study? Or do you mean you looked at even the longitudinal data cross-sectionally to facilitate comparison?

*Yes, cross-sectional refers to cull data i.e. only has one time point intrinsically. However, mice entering in to the longitudinal study were also culled if captured 4 weeks later and therefore represent a subset of the cross sectional data. We have added section to our Methods as detailed above to better explain this, as well as a supplementary figure detailing the trapping schedule at each grid (Lines 453-467).*

L378: A fair hypothesis, but the data may not necessarily reflect that given that egg burden was not a good predictor of worm burden. The authors should discuss this discrepancy at least briefly, explaining why parasite fecundity affects host health.

*We note in our discussion that egg counts are not a strong indicator of worms burdens (line 407,* The fact that egg counts are not a strong indicator of worm burdens limits the usefulness of these analysis if egg counts are used as a proxy for worm burden, and should be kept in mind for other field studies using egg burdens as an indicator of infection levels*. We have added this as a caveat to our discussion of increasing body condition correlating with a decrease in egg burden being an indicator of fitness cost (line 413):*

While taking these limitations into account, alongside the fact that egg shedding can vary throughout the day, we suggest that repeated measures of faecal egg burden still provide a valuable insight into parasite fitness.

The increase in body condition concomitant with a decrease in faecal egg burdens over time indicates that infection may confer some level of fitness cost in the mice, akin to studies showing measurable health benefits of reduced egg shedding (58, 60, 61).

L405: Please include trapping effort (trap-nights, interval between trapping sessions, etc.) if the information is available. Did the longitudinal lab experiment occur at the same time as these sampling sessions?

*We have included a supplementary figure detailing the trapping schedule, and clarified trapping effort in the text (Lines 453-467) as detailed above. The lab experiment did not occur at the same time as these sampling sessions for logistical reasons.*

L451: Please explain at least briefly (salt flotation, McMaster slides, etc).

*We have clarified our egg counting protocol in the methods (lines 592-601).*

L454: Was the isolation of lymph nodes done in situ on the island? Or were samples transported somewhere else, and if so, what were the storage conditions and storage duration of samples?

*Dissection of wild mice and lymph node extraction, culturing and supernatant collection was done in situ on the island. Culture supernatants were stored at -80 degrees and transported back to the laboratory in Manchester on dry ice, prior to cytokine analysis in the lab. To clarify this, we have added the following sentence to the Materials and Methods section, lines* *526-531.*

Mesenteric lymph node cells were cultured in situ (on the Isle of May for wild mice, in the laboratory for laboratory mice) …

Supernatants from wild mice were stored at -80 degrees on the island prior to transport on dry ice to the University laboratories, where cytokine analysis was performed using the same assay and acquisition setup as for laboratory mice.

L571: This is the first time that recapture is mentioned. Were any of the wild mice culled at first trapping? Additionally, why did these data led the authors to believe that the standard aging protocol was inaccurate? Looks like the authors undertook 4 months of trapping each year, so perhaps the specific issue was something like: mice captured repeatedly for 4 months were estimated to be just 2 months old based on the final mass of their eye lens alone?

*We apologise for our lack of clarity in terms of our experimental design and we have now inserted the following text in to our Methods under Mice (Lines 453-467) – also included in our response to the referees comment (their reference to L250):*

Mice captured from November to December in 2018 and from September to December 2019, and which had been previously trapped, were culled (n=214). Another 58 untagged mice caught during culling sessions were also culled, where fieldwork capacity allowed. All culled mice formed our ‘Cross-Sectional’ set (n=272), and were used for detailed immunological and histological analyses. Of these culled mice, 91 animals had been captured 30 days previously (recaptures) and so also entered into our ‘Longitudinal’ study, allowing us to investigate changes in individuals over time. Across the two years, overall recapture rate was 40% within one month.

*Regarding the eye lens weight age estimate, the reviewer is correct in that we found that a number of animals were deemed several weeks younger than was physically possible, based on the assumption that any caught animal was at least 2-3 weeks old (post-weaning).*

L597: Linear or Generalized Linear mixed model?

*We have clarified this now in the methods section as per below:*

*(Lines 674-676)* For longitudinal analysis, linear mixed models were used to assess the influence of changing infection status and antibody levels on gut responsiveness and changes in body condition while controlling for inter-individual variation.

***Response to Reviewer 3***

*We thank reviewer 3 for their helpful review and the interesting and engaged comments they have made. Our response to each comment is below, with any new content indicated as appropriate. We have also taken into account the advice of the editor that no additional experimentation is required.*

Fig. 2F – I am not in favor of the color oval overlays. Personally, I would prefer to just see the individual colored data points – this more than makes the point the authors are emphasizing.

*We have revisited our Figure 2F and looked carefully at the colour schemes and the use of colour ovals. With due respect to the referee we actually disagree and believe the figure as is presents the data in the most accessible manner.*

I was not able to download the hi-res tif of Fig 3, but it does not appear that any of the graphs in Fig 3C specifically show proportions of Tem, even though this is referred to in the text (line 216). I appreciate the desire to not clutter the figure with labels, but it is not always clear which data in the figure are being referred to in the text. Suggest rethinking this labeling scheme.

*We agree it is not very easy to find the relevant graph, so we have added boxes 1-6 to Figure 3C and refer to these in the results text in the appropriate place*.

Line 238 – “The other variables including sex, body condition, and month of capture were not able to explain cytokine expression patterns.” But what about infection with other parasites? Do any of the immunological parameters (not just cytokines) correlate with Calodium and/or Syphacia infection? Please specifically address this point. I was puzzled it wasn't addressed further. One would speculate that Calodium infection in particular could cause systemic immunological effects.

*The referee raises an interesting point, which in future, with bigger sample sizes, may be able to be addressed and we would be very interested in pursuing further. However, there are limited diagnostic tools available for Calodium to determine infection levels and immunological consequences; we acknowledge that the visual assessment of the liver is a crude method of assessing Calodium infection levels. With the current study population of 272 animals, taking into account co-infection and the severity of co-infection would not provide enough power to come to statistically significant conclusions. We have now included a comment regarding potential immunomodulation and the lack of Calodium-specific tools in the results section (lines 176-178)*

Whilst immunomodulation by *Calodium* is likely, we did not carry out further parasite-specific assays due to the scarcity of diagnostic tools for this parasite (35).

Does the Isle of May mouse population not harbor any cestodes, either gut-dwelling adults, or metacestodes elsewhere in the body? Just curious. One of the "benefits" perhaps of an isolated population?

*We occasionally find tapeworms (Hymenolepes spp) but these are rare in comparison to Trichuris. Interestingly, the Isle of May mouse population appears to have an unusually high prevalence of Trichuris (79%) compared to for example mainland mice (e.g. 13%; Zhang H, Bednář L, Heitlinger E, Hartmann S, Rausch S. Whip- and pinworm infections elicit contrasting effector and distinct regulatory responses in wild house mice. Int J Parasitol. 2022 Jul;52(8):519-524. doi: 10.1016/j.ijpara.2022.03.006. Epub 2022 May 6. PMID: 35533731).*

Along those same lines, and without knowing anything about this specific population, it would be helpful to know if there are any other characteristics of the island population that might be relevant immunologically, for example, has the population been subject to any genetic bottlenecks that might have reduced genetic diversity relative to mouse populations elsewhere?

*It is unknown when exactly mice were introduced to the Isle of May, but they have likely been brought on frequently during the various inhabited periods on the island, with pilgrimage to the island being recorded since the 5^th^ century AD (*[*https://isleofmaynnr.wordpress.com/2017/05/19/1000-years-of-history-on-the-may/*](https://isleofmaynnr.wordpress.com/2017/05/19/1000-years-of-history-on-the-may/)*). The first written mention appears in the 19^th^ century in a lighthouse keeper’s diary (Ecclesiological Notes on Some of the Islands of Scotland by T S Muir, 1885). Additionally, there has been a re-introduction experiment performed by Berry et al. in 1982, releasing 77 wild house mice from the island of Eday (Orkney, Scotland, UK). Follow-up studies found that their genome had introgressed into the whole island population within half a year (Berry et al., Journal of Zoology 1991, doi.org/10.1111/j.1469-7998.1991.tb04329.x). Since the establishment of the Isle of May as a Nature Reserve in 1956, it is likely that accidental introductions have been reduced due to the effort to maintain the island rat-free for the safety of the ground-nesting seabirds. Every winter, the population crashes from an estimated few thousands to an estimated few hundred individuals (Triggs, Journal of Zoology 1991, doi.org/10.1111/j.1469-7998.1991.tb03828.x), so there is a high selection pressure.*
